# Designing strategies of Pingyao lacquerware tourist souvenirs based on tourists’ demand

**DOI:** 10.1371/journal.pone.0305662

**Published:** 2024-07-29

**Authors:** Xing Zhang, Jasni Dolah

**Affiliations:** Visual Communication Design Department, School of Arts, Universiti Sains Malaysia, Penang, Malaysia; AlloTec Bio, UNITED STATES

## Abstract

The continuous development of the economy and the constant improvement in living standards have led tourists to have higher expectations for the entire travel experience. However, outdated Pingyao lacquerware tourism souvenirs have struggled to attract tourists’ attention and cannot better meet their demands. It is worth considering whether analyzing tourists’ demands could solve the current issues with lacquerware tourism souvenirs. To address this question, tourists in the Pingyao region were surveyed, and semi-structured interviews and questionnaires as research methods. Initially, semi-structured interviews were conducted with tourists in the Pingyao region to obtain the initial demand indicators for Pingyao lacquerware tourism souvenirs. Based on a literature review and expert summaries, 21 demand indicators were selected for the KANO model questionnaire, and 400 tourists were surveyed using stratified sampling. Data analysis revealed that of the 21 demand indicators evaluated by tourists, 5 as must-be quality, 7 as one-dimensional quality, 5 as attractive quality, and 4 as indifferent quality. The results of this study indicate that the demand attributes evaluated are related to tourists’ satisfaction levels, and the lack of these attributes could lead to dissatisfaction with lacquerware tourism souvenirs. Tourists have shown a strong demand for appearance and a desire for functional diversification in lacquerware souvenirs, with higher expectations for regional and commemorative aspects. Due to the uniqueness of lacquerware materials, tourists have highly prioritized safety and environmental friendliness. Combining the KANO model and AHP have better-assisted researchers in identifying the quality types and importance of tourist demands, providing more targeted insights for designing Pingyao lacquerware tourism souvenirs.

## Introduction

Today’s rapidly evolving social and economic landscape makes people’s lives increasingly fast-paced. Tourism is highly regarded as a means of stress relief. Tourism shopping has always been a part of the tourism industry’s development. Tourist souvenirs carry regional culture and interpretations and creations of regional culture. Tourist souvenirs have become one of the most prominent targets in tourism consumption, generating billions of dollars in revenue for destinations worldwide yearly [1]. They are an integral part of tourism, and purchasing souvenirs is considered an everyday holiday activity [2]. Souvenirs can trigger positive trip memories [3] and allow tourists to express themselves [4]. Many tourists feel their travel experience would be incomplete without purchasing souvenirs for themselves or as gifts for friends and family [5]. Therefore, the design of tourist souvenirs is of great importance, as it plays a positive role in promoting local economic development and promoting tourist cities and culture [6].

As an integral part of Pingyao Ancient City’s tourism shopping market, Pingyao lacquerware tourist souvenirs play a crucial role in developing Pingyao’s tourism industry. Pingyao Ancient City, one of China’s four well-preserved ancient cities, has rich tourism resources [7]. In 1997, Pingyao Ancient City was inscribed in the UNESCO World Heritage List, and tourism became a pillar industry that rapidly expanded. Currently, within Pingyao Ancient City, tourist souvenirs primarily include lacquerware, handmade paper-cut art, Liu He Tai pillows, and handmade cloth shoes, with Pingyao lacquerware tourist souvenirs occupying the largest market share [8]. However, in the current tourism development process in Pingyao, the design of Pingyao lacquerware souvenirs has become outdated and cannot better attract or suit the demands of tourists [9]. Simultaneously, awareness of souvenir development remains low, with the majority of Pingyao lacquerware souvenirs manufactured in local workshops or studios by local craftsmen. There are few designers engaged in lacquer tourist souvenirs industry, and the products still follow the traditional patterns and patterns to a large extent, lacking innovation and regional cultural characteristics [10]. For tourists, acquiring tangible reminders of specific times and places in souvenirs and handicrafts is common [11]. Hence, tourists have a fondness for tourist souvenirs that are associated with and unique to the destination [12, 13], as many are not interested in buying inauthentic souvenirs because they want something unique and tied to the destination [5, 14–16]. In other words, souvenirs should reflect the uniqueness of the place, representing and being closely associated with it [17].

Tourists’ demands refer to something that tourists feel is lacking in psychology and physiology [18], they have various demands during their travel activities, which drive their tourism activities [19]. Any cultural resource that aspires to be a vibrant and dynamic "present tense" or "present continuous tense" must be related to the demands of the public today [20]. An essential factor in measuring the value of souvenirs is the quality of tourist souvenirs and their ability to meet various user demands [21]. However, actual markets often overlook tourists’ true demands during travel [22]. Khalid and Abdulla [23] argues that understanding tourists’ demands is crucial for souvenir design. It is necessary to understand the demands of tourists through investigation and obtain reference data for design guidance, so as to improve the satisfaction of tourists. So, tourists’ demands are a core factor in the design of tourist souvenirs [24]. Therefore, designing lacquerware tourist souvenirs that are regionally distinctive and capable of meeting tourists’ demands is particularly attractive. Furthermore, lacquerware tourist souvenirs serve as a means of communication with tourists [25]. Lacher suggests that tourist souvenirs are vehicles for cultural dissemination, promoting regional economic development while promoting the region’s image [26]. Therefore, well-designed lacquerware tourist souvenirs positively promote Pingyao’s local economic development and cultural dissemination [27] and help enhance tourists’ impressions of Pingyao Ancient City [8]. In brief, for improving the value of lacquer souvenirs, it is necessary to optimize the design strategy of lacquer souvenirs.

Therefore, this study used semi-structured interviews and the Kano questionnaire method to explore tourists’ demands for Pingyao lacquerware tourist souvenirs, ultimately determining design preferences for souvenirs. The Kano model effectively identifies design demands that affect product satisfaction [28]. Initial data on Pingyao tourists’ demands were obtained through semi-structured interviews. Based on the interview results, relevant data were organized, and with input from a literature review and expert opinions, tourist demands for Pingyao lacquerware souvenirs were determined to ensure the accuracy and professionalism of the questionnaire design. In this study, design preferences were determined through Kano and Analytic Hierarchy Process (AHP), leading to the establishment of design strategies for lacquerware souvenirs, thereby enhancing tourist satisfaction with these souvenirs. As a result, tourists are investigated and data is analyzed in order to realize the design of lacquer tourist souvenirs based on tourists’ demands, improve tourists’ desire and satisfaction in purchasing souvenirs, and therefore enhance economic benefits and cultural promotion.

## Methods

This study uses semi-structured interviews with tourists to ensure the professionalism and reliability of gathering tourist demands through a literature review and Delphi method interviews with experts. The Kano model can be used to precisely identify which product design demands will enhance tourist satisfaction [28]. By combining the Kano model with the AHP, it is possible to accurately determine tourist demands and their importance rankings for Pingyao lacquerware tourist souvenirs [29], thereby providing valuable references for the design of lacquerware tourist souvenirs. The data collection of this study was approved by the Ethics Review Committee of Universiti Sains Malaysia with the approval number USM/JEPeM/PP/23120989, and the written informed consent of the participants was obtained.

### Study area and target population

Data were collected from tourists in Pingyao Ancient City, Shanxi Province, China. Pingyao Ancient City, located in the central part of Shanxi Province, is the most well-preserved ancient county in China, featuring rich and unique architectural, religious, commercial, and folk cultural elements. In 1986, the State Council named Pingyao Ancient City as the second batch of National Historic and Cultural Cities. In 2015, it was approved as a national 5A-level tourist attraction. Pingyao lacquerware, a representative city card of Pingyao, was listed as a national intangible cultural heritage in 2006, becoming one of China’s four significant lacquerwares alongside Beijing, Yangzhou, and Fujian lacquerware. The target population for data collection were tourists and experts. To obtain initial data on tourists’ demand for Pingyao lacquerware souvenirs, 18 tourists were interviewed in semi-structured interviews. Hennink et al. found in a 25-person interview study that approximately 90% of the concepts could be obtained after the ninth interview, and half of the concepts were mentioned in the first interview. However, to capture more than 95% of the information, interviews with 15 or more people were required. From the actual sequence of interviews, starting from the 16th interviewee, four consecutive individuals provided no new information, indicating that the range of codes or themes had been essentially determined, allowing for the determination that the study had reached code or theme saturation [30]. Therefore, 18 tourists with a particular understanding of Pingyao lacquerware tourism souvenirs were interviewed to determine the demand indicators for the questionnaire, which 15 experts then validated. Krejcie & Morgan suggested that the total population size affects the sample size; for a total population (N) > 1,000,000, a sample size of 384 or more is appropriate [31]. According to data released by the Pingyao Culture and Tourism Bureau in the first half of the year, Pingyao Ancient City had received 1.027 million visitors by the end of March. Therefore, selecting 400 tourists as the sample size for the subsequent questionnaire survey is appropriate.

### Sample selection and exclusion criteria

The selection criteria for tourists are: 1) Have visited Pingyao Ancient City; 2) Understand Pingyao lacquerware (at least know what Pingyao lacquerware is) or have purchased Pingyao lacquerware. The exclusion criteria are: 1) Individuals under 18 years old. In China, according to the Civil Code, people under 18 who have not formed a complete cognition of things and do not possess the capacity for economic independence; 2) Individuals with intellectual disabilities and lacking independent judgments. The selection criteria for experts are: 1) Engaged in the tourism-related service industry; 2) Involved in the lacquerware industry; 3) Have at least 5 years of design work experience and have achieved specific work results. The exclusion criteria are individuals who are uninterested in this study and lack enthusiasm.

### Questionnaire design

The questionnaire design for this study is based on the KANO model questionnaire. The KANO model is a valuable tool for categorizing and prioritizing user demands, and it was invented by Professor Noriaki Kano of the Tokyo Institute of Technology in 1984. It classifies customer demands into five categories based on the relationship between different demands and customer satisfaction: i) Must-be quality, which are attributes or features that customers consider a product "must have." Failure to meet these demands results in customer dissatisfaction, and meeting them might not necessarily result in satisfaction. ii) One-dimensional quality, where satisfaction significantly increases if these demands are met or performed well, and dissatisfaction significantly increases if they are not met or performed poorly. iii) Attractive quality, where meeting these demands, even if not perfectly, results in very high customer satisfaction, but the absence of these does not necessarily result in dissatisfaction. iv) Indifferent quality refers to features that do not impact the customer experience, whether provided or not. v) Reverse quality, a demand that customers do not have, and provide these can decrease satisfaction. Additionally, each question in the KANO questionnaire consists of positive and negative questions to understand and categorize customers’ perceptions of product demands. In this questionnaire, positive questions measure tourists’ evaluations of lacquerware tourism souvenirs with certain features, while negative questions measure tourists’ evaluations of the absence of these features. The questionnaire design is as follows (Table 1):

**Table 1:** KANO questionnaire.

Question:	Very satisfied	Should be like this	It doesn’t matter	Tolerable	Dissatisfied
How do you feel if there is this feature in the product?					
How do you feel if there is not this feature in the product?					

### Data analysis techniques

This study utilized SPSS for data analysis of the KANO model questionnaire. The model primarily evaluates user satisfaction with elements related to systems or services [32–34]. This analytical method suitable for the research objectives of this questionnaire. The KANO model requires the calculation of Better-Worse coefficients to analyze how much a feature can increase satisfaction or mitigate displeasure.

The specific formulas for calculating the Better-Worse coefficients, as proposed by Berger, are as follows:

$$Better/SI=\frac{\left(O+A\right)}{\left(M+O+A+I\right)}$$

$$Worse/DSI=–1\times \frac{\left(O+M\right)}{\left(M+O+A+I\right)}$$

- When the absolute values of the Better and Worse coefficients are more significant than 0.5, the current demand is considered one-dimensional quality.
- When the absolute values of both the Better and Worse coefficients are less than 0.5, it is considered indifferent quality.
- When the Better coefficient is more significant than 0.5, and the absolute value of the Worse coefficient is less than 0.5, it is considered attractive quality.
- When the Better coefficient is less than 0.5, and the absolute value of the Worse coefficient is more significant than 0.5, it is considered must-be quality.

A quadrant chart is constructed by calculating the better-worse coefficients for each feature. Within the same quadrant, features with higher Better values are generally prioritized.

Additionally, when using the KANO model, it is expected to combine it with other management tools such as Quality Function Deployment (QFD), the Theory of Inventive Problem Solving, and AHP [35]. Currently, the KANO model is limited in its application to other management tools to enhance the quality of tourism souvenirs in China, which may become a research focus in the future. Due to its limitations, the KANO model cannot accurately determine the importance of each user demand, potentially leading to deviations in design priorities during subsequent product development [36].

In the 1970s, American operations researcher Saaty introduced the Analytic Hierarchy Process (AHP). This multi-criteria decision-making method combines qualitative and quantitative analyses to determine the importance of various elements. It is known for its simplicity and objectivity. Applying AHP can accurately determine the weights of user demands in the KANO model [37]. Therefore, this study combines KANO model analysis with AHP to calculate demand weights more scientifically, compensating for the statistical shortcomings of the KANO model to some extent. AHP has four weight calculation methods: geometric mean, arithmetic mean, eigenvalue method, and least squares method. In this study, the geometric mean method was chosen, with the following steps:

1. Multiply the elements in each row of the judgment matrix, and *b*_*ij*_ represents the importance scale of i to j, that is
$$M_{i}=\prod_{j=1}^{n}b_{ij},(i=1,2\ldots ,n)$$
2. Calculate the geometric average of *M*_*i*_, that is
$$\bar{W_{i}}=\sqrt[{n}]{M_{i}},(i=1,2\ldots ,n)$$
3. Calculate $\bar{W_{i}}$ in a standardized way, that is
$$W_{i}=\frac{\bar{W_{i}}}{\sum_{i=1}^{n}\bar{W_{i}}},(i=1,2\ldots ,n)$$
4. Consistent test, find the maximum characteristic root *λ*_*max*_, where B is the known judgment matrix, n is the order, and W represents the weight column vector, that is
$$\lambda_{max}=\frac{1}{n}\sum_{i=1}^{n}\frac{{\left(B\cdot W\right)}_{i}}{W_{i}}$$
5. Consistency index, that is
$$CI=\frac{\lambda_{max}-n}{n-1}$$
6. Consistency ratio Cr; RI represents the proportional coefficient, and its value is related to the order n of the judgment matrix, that is
$$CR=\frac{CI}{RI}$$
  If Cr < 0.1, the consistency test is satisfied. Otherwise, the judgment matrix is modified.

### Data collection

The data collection periods were selected to coincide with Chinese holidays. There are many holidays throughout the year in China, such as the Spring Festival, Tomb-Sweeping Day, Labor Day, Dragon Boat Festival, National Day, etc. During each holiday, Chinese citizens typically have corresponding time off, and most people choose to travel, facilitating the data collection process.

Firstly, a stratified random sampling method was used to conduct semi-structured interviews with 18 tourists who had some understanding of Pingyao lacquerware tourism souvenirs (at least knowing what Pingyao lacquerware is). These interviews were designed to capture the tourists’ perspectives and demands for Pingyao lacquerware tourist souvenirs. Each interview with a tourist did not exceed 30 minutes [38], and the data collection was completed within six days. Details of the interviewed tourists are provided in Table 2. After the interviews, data transcription was carried out promptly, and data analysis was performed using NVivo. Combining this analysis with the relevant literature review, tourist demands for Pingyao lacquerware tourist souvenirs were obtained, and a preliminary questionnaire item pool was established.

**Table 2:** Tourists’ information.

No.	Gender	Age	Educational	Occupation
1	Female	30–45	Undergraduate	Public servant
2	Female	46–60	High School	Worker
3	Female	18–29	Undergraduate	Student
4	Male	18–29	Undergraduate	Designer
5	Female	60 above	Undergraduate	retired
6	Female	30–45	Postgraduate	College teacher
7	Female	46–60	Undergraduate	Housewife
8	Female	30–45	Undergraduate	Clerk
9	Male	46–60	Undergraduate	Public servant
10	Male	30–45	Postgraduate	Ground crew
11	Female	30–45	Postgraduate	Doctor
12	Female	30–45	Undergraduate	Wood craftsman
13	Male	18–29	Undergraduate	Interior designer
14	Female	46–60	Undergraduate	Salesman
15	Male	30–45	Undergraduate	Public servant
16	Female	60 above	High School	retired
17	Male	18–29	Postgraduate	Student
18	Male	30–45	PHD	College teacher

Then the convenience sampling method was used to select 15 experts from Beijing, Shanxi, Fujian, and Jiangsu as the participants for the Delphi study. The criteria for expert selection were as follows: 1) Engagement in the tourism-related service industry, 2) Involvement in work related to the lacquerware industry, 3) At least five years of design work experience with significant accomplishments, and 4) A high level of enthusiasm for this research [39]. Details of the selected experts can be found in Table 3. This study conducted two rounds of Delphi expert consultations and performed statistical analysis on the experts’ positivity coefficient, authority level, and Kendall harmony coefficient (Kendall W), as presented in Tables 4 and 5. Before this, the study’s purpose, content, and research process were explained to the experts.

**Table 3:** Experts’ information.

Basic information	The first round of consultation (n = 13)	The second round of consultation (n = 12)
**Gender**		
Male	8/13	7/12
Female	5/13	5/12
**Age**		
35–45	4/13	4/12
46–55	6/13	5/12
56–65	3/13	3/12
**Working life(Years)**		
5~15	2/13	2/12
16~25	8/13	7/12
>25	3/13	3/12
**Working area**		
Tourism	3/13	3/12
Related to lacquerware	6/13	5/12
Design	4/13	4/12

**Table 4:** Authority coefficients of experts.

Number of consultations	Cs	Ca	Cr
First	0.870	0.802	0.836
Second	0.858	0.776	0.817

**Table 5:** Coordination degrees of expert opinions.

Index	First round of consultation		
	No.	Kendall’s W	P
First	26	0.673	<0.001
Second	21	0.723	<0.001

The response rates for the two rounds of consultations were 87% and 92%, indicating a high level of enthusiasm among the expert group. The authority coefficient (Cr) of expert opinions in the first and second rounds was 0.836 and 0.817, respectively, suggesting a high level of authority among the experts and reliable consultation results. The Kendall harmony coefficient values range from 0 to 1, with larger values indicating better agreement. Table 5 demonstrates that Kendall’s W values for primary indicators in the first round is 0.673, in the second round, the Kendall’s W values is 0.723, indicating good agreement among the experts. This method enhanced the completeness and scientific nature of the content. After aggregating and ranking tourist demands, the final results included four primary and 21 secondary items, as shown in Table 6.

**Table 6:** Tourists’ demand.

Dimension	Index	Demand Point	Demand Point Description
Appearance Demands	T7	Shape	Beautiful shape
	T8	Pattern	Nice pattern
	T9	Color	Nice color matching
	T10	Material	Many types of materials
	T11	Craft	Exquisite craft
Production Demands	T12	Price	Reasonable price
	T13	Type	Rich types of products
	T14	Quality	Quality can be guaranteed
	T15	Portable	Lightweight and easy to carry
	T16	Function	With multiple functions
	T17	Culture	With cultural taste
	T18	Series	Have series
	T19	Packing	Good packing
Emotional Demands	T20	Brand	Good brand
	T21	Interesting idea	Interesting idea
	T22	Commemorative significance	Great commemorative significance
	T23	Regional	Obvious regional characteristics
	T24	Gift	Suitable as a gift
	T25	Collection	Suitable for collection
Extra Demands	T26	Safe	High security
	T27	Environmental protection	Environmentally friendly

Simultaneously, based on the questionnaire items established through expert consultations, a Kano attribute questionnaire regarding tourist demands for lacquerware souvenir items was developed. Additionally, to ensure the reliability and validity of the final questionnaire, a pilot survey of Kano attribute questionnaires for lacquerware souvenir item demands was conducted among 40 tourists in Pingyao Ancient City.

Finally, a formal questionnaire survey was conducted with 400 tourists. Participants needed approximately 5–10 minutes to complete the questionnaire. The data collection was completed within ten days. The questionnaires were administered anonymously via scanning QR codes and filling out the survey online using the Wenjuanxing tool. Wenjuanxing is China’s largest online survey platform, allowing users to create and distribute questionnaires. Additionally, Wenjuanxing provides convenient data collection features. After the questionnaires were published, researchers could collect participants’ responses in real time, enabling them to quickly access survey results [40].

## Results and analysis

### Reliability and validity analysis

In this study, 40 tourists were pre-tested by convenient sampling method to test the reliability and validity of the questionnaire.

Reliability is an assessment of the degree to which assessment results are reliable. This study used Cronbach’s α to test the reliability of the questionnaire. A Cronbach’s α value greater than 0.7 is considered acceptable. A Cronbach’s α between 0.7 and 0.8 indicates the reliability is good, and between 0.8 and 0.9 suggests the reliability is quite good. A Cronbach’s α greater than 0.9 indicates excellent reliability. This study primarily used SPSS to assess the reliability, and the reliability test results for each scale are shown in Table 7. The results indicate that Cronbach’s α for both positive and negative questions is more significant than 0.7, which suggests reasonable reliability. Additionally, exploratory factor analysis was conducted using SPSS. A Kaiser-Meyer-Olkin (KMO) test and Bartlett’s sphericity test were performed to evaluate data validity. In the empirical study, KMO values above 0.7 indicate good validity, KMO values between 0.6 and 0.7 indicate moderate validity, and Bartlett’s sphericity test should have a significance level less than 0.05. The results indicate that KMO values for both positive and negative questions are greater than 0.7. Bartlett’s sphericity test results are significant (p < 0.001), suggesting good data validity, as shown in Tables 8 and 9.

**Table 7:** KANO questionnaire reliability results.

Question	Cronbach’s α	Number
Positive question	0.781	21
Negative question	0.755	21

**Table 8:** Positive question KMO and Bartlett test.

Sample suitability quantity		0.733
Bartlett’s sphericity test	The last chi-square read	7109.799
	Freedom	210
	Significance	0.000

**Table 9:** Negative question KMO and Bartlett test.

Sample suitability quantity		0.714
Bartlett’s sphericity test	The last chi-square read	6163.418
	Freedom	210
	Significance	0.000

### Data analysis

Considering the constraints and uncontrollable factors during the questionnaire collection process, this study distributed 20% more questionnaires to ensure that at least 400 valid questionnaires could be obtained. In total, 480 questionnaires were distributed, and 419 valid questionnaires were collected (including 37 with completion times less than 90 seconds, 13 indicating "do not know" or "never purchased," and 11 from participants under 18 years old, all of which were considered invalid), resulting in a response rate of 87.3%.

The questionnaire data were organized and analyzed using the KANO model. By calculating Better and Worse coefficients, each tourist’s attributes were determined. If the absolute values of Better and Worse are greater than 0.5, it represents a One-dimensional quality (O). If the absolute values of Better and Worse are less than 0.5, it indicates an Indifferent quality (I). When Better is greater than 0.5, and the absolute value of Worse is less than 0.5, it signifies an Attractive quality (A). Conversely, when Better is less than 0.5, and the absolute value of Worse is greater than 0.5, it represents a Must-be quality (M). The statistical results are presented in Table 10.

**Table 10:** Analysis result of tourist’s demand.

Index	Dimension	A (%)	O(%)	M(%)	I(%)	Better	Worse	Attribute
T7	Appearance Demands	19.81	26.01	44.39	8.83	0.4627	-0.7108	M
T8		26.01	17.42	44.39	10.50	0.4417	-0.6286	M
T9		9.31	22.20	46.78	19.09	0.3235	-0.7083	M
T10		26.25	14.08	22.91	36.75	0.4033	-0.3699	I
T11		29.59	30.79	27.68	10.98	0.6096	-0.5904	O
T12	Production Demands	25.06	32.46	26.01	13.84	0.5907	-0.6005	O
T13		28.40	26.49	36.75	7.40	0.5542	-0.6386	O
T14		13.13	23.63	50.84	12.41	0.3675	-0.7446	M
T15		53.46	24.58	16.95	2.39	0.8015	-0.4265	A
T16		41.53	29.12	24.11	3.10	0.7220	-0.5439	O
T17		33.41	37.47	26.49	1.67	0.7157	-0.6458	O
T18		17.18	22.20	25.06	34.84	0.3966	-0.4760	I
T19		39.62	19.81	24.58	14.08	0.6058	-0.4526	A
T20	Emotional Demands	44.39	18.85	29.12	5.49	0.6463	-0.4902	A
T21		52.27	15.51	22.20	8.59	0.6877	-0.3826	A
T22		34.84	34.61	21.00	6.92	0.7132	-0.5711	O
T23		33.89	35.08	22.91	5.97	0.7049	-0.5927	O
T24		45.35	16.95	24.82	11.46	0.6320	-0.4237	A
T25		26.01	13.84	21.96	38.19	0.3986	-0.3580	I
T26	Extra Demands	25.78	14.08	23.87	36.28	0.3986	-0.3795	I
T27		10.26	21.96	54.18	13.60	0.3222	-0.7613	M

**Fig 1:**
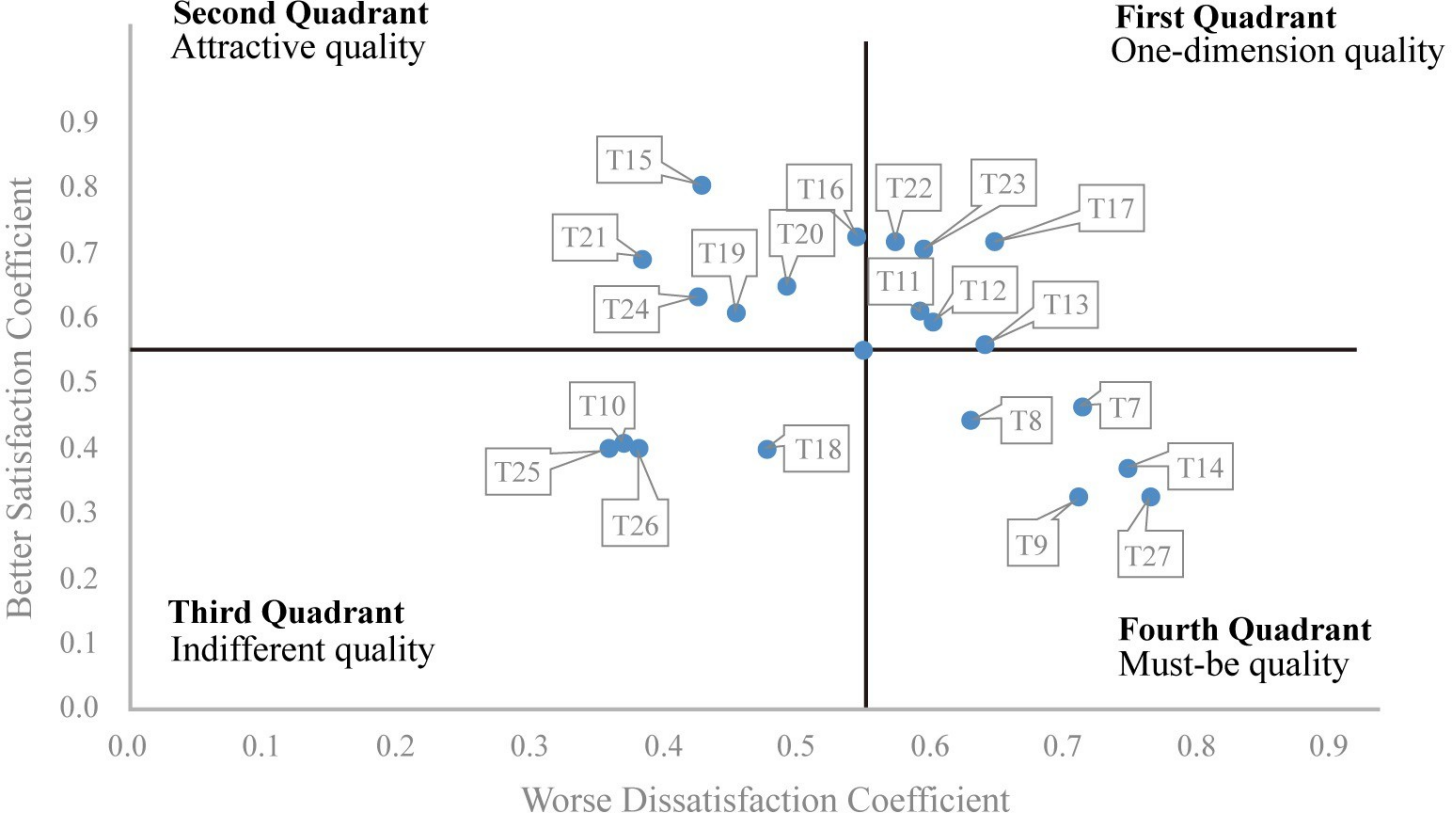
The quadrant distribution map of tourist’s demands. NOTE: This figure shows the quadrant distribution of four qualities demanded by tourists, which is obtained according to the results of Kano analysis.

By calculating the Better-Worse values, researchers can construct a quadrant chart, providing a more intuitive observation of the impact of meeting a specific demand on increasing tourist satisfaction or eliminating dissatisfaction. In Kano model theory, better values are typically positive, indicating that user satisfaction will increase if a product offers a particular feature or service. A more considerable value suggests a more substantial effect on enhancing satisfaction and a faster increase in user satisfaction. Conversely, Worse values are usually negative, signifying that user satisfaction will decrease if a product does not provide a specific feature or service. A more significant value represents a more pronounced effect on reducing satisfaction and a faster decline in user satisfaction. Based on the Better-Worse values of the 21 tourist demands, Fig 1 illustrates the quadrant chart.

1. Demands in the first quadrant belong to One-dimensional quality, with relatively high absolute values for both Better and Worse. Tourist satisfaction varies significantly, meaning that if the lacquerware tourism souvenirs offer or improve services or features falling into this quadrant, tourist satisfaction will increase. Conversely, if they do not, tourist satisfaction will decrease.
2. Demands in the second quadrant belong to Attractive quality: Better has a high absolute value, while Worse has a low absolute value. If lacquerware tourism souvenirs do not provide services falling into this quadrant, tourist satisfaction will not decrease. However, tourist satisfaction will significantly increase if they do provide these services.
3. Demands in the third quadrant are considered Indifferent quality: both Better and Worse have low absolute values. Tourist satisfaction remains unchanged, as tourists are indifferent to whether these services or features are provided or not by lacquerware tourism souvenirs.
4. Demands in the fourth quadrant fall under Must-be quality: Better has a low absolute value, while Worse has a high absolute value. When lacquerware tourism souvenirs offer this feature, tourist satisfaction will not increase. However, tourist satisfaction will significantly decrease when this feature is not provided.

Additionally, tourists have varying sensitivities to different demands within the same dimension. To assess tourist sensitivity to each demand, researchers use AHP for pairwise comparisons, constructing comparison matrices to calculate the weight values for each demand. This process achieves a hierarchical ranking of lacquerware tourism souvenir demands.

Initially, an indicator evaluation system is built based on the KANO results (Table 11). In this system, Indifferent quality imply that respondents have a neutral attitude toward the demand. Therefore, T10, T18, T25, and T26 are not analyzed. Using the evaluation system (Table 11), researchers construct judgment matrices to compare each demand indicator, ranking the various demands. According to the previously mentioned calculation formula, the results are as follows (Tables 12–16).

**Table 11:** Evaluation system for KANO demands indexes of lacquerware tourist souvenirs.

Dimension	Index
Must-be quality	T7
	T8
	T9
	T14
	T27
One-dimensional quality	T11
	T12
	T13
	T16
	T17
	T22
	T23
Attractive quality	T15
	T19
	T20
	T21
	T24

**Table 12:** Weight of first-level demand index.

	Must-be quality	One-dimensional quality	Attractive quality	Weight
Must-be quality	1	2	3	0.5278
One-dimensional quality	1/2	1	3	0.3325
Attractive quality	1/3	1/3	1	0.1396

**Table 13:** Weight of secondary indicators of must-be demand.

	T7	T8	T9	T14	T27	Weight
T7	1	2	3	1/2	1/3	0.1693
T8	1/2	1	2	1/2	1/3	0.1183
T9	1/3	1/2	1	1/3	1/4	0.0720
T14	2	2	3	1	1/2	0.2422
T17	3	3	4	2	1	0.3982

**Table 14:** Weight of secondary indicators of one-dimensional quality.

	T11	T12	T13	T16	T17	T22	T23	Weight
T11	1	2	2	1/5	1/3	1/2	1/2	0.0797
T12	1/2	1	1	1/4	1/3	1/2	1/3	0.0577
T13	1/2	1	1	1/5	1/4	1/2	1/2	0.0568
T16	5	4	5	1	2	2	3	0.3230
T17	3	3	4	1/2	1	2	3	0.2290
T22	2	2	2	1/2	1/2	1	2	0.1430
T23	2	3	2	1/3	1/3	1/2	1	0.1107

**Table 15:** Weight of secondary indicators of attractive quality.

	T15	T19	T20	T21	T24	Weight
T15	1	4	3	2	3	0.3893
T19	1/4	1	1/3	1/4	1/2	0.0664
T20	1/3	3	1	1/2	2	0.1655
T21	1/2	4	2	1	3	0.2721
T24	1/3	2	1/2	1/3	1	0.1067

**Table 16:** Weight result of index system.

Dimension	Weight	Index	Weight	Comprehensive weight	Sequence
Must-be quality	0.5278	T27	0.3982	0.2102	1
	0.5278	T14	0.2422	0.1279	2
	0.5278	T7	0.1693	0.0894	4
	0.5278	T8	0.1183	0.0624	6
	0.5278	T9	0.0720	0.0380	10
One-dimensional quality	0.3325	T16	0.3230	0.1074	3
	0.3325	T17	0.2290	0.0762	5
	0.3325	T22	0.1430	0.0476	8
	0.3325	T23	0.1107	0.0368	11
	0.3325	T11	0.0797	0.0265	12
	0.3325	T12	0.0577	0.0192	14
	0.3325	T13	0.0568	0.0189	15
Attractive quality	0.1396	T15	0.3893	0.0544	7
	0.1396	T21	0.2721	0.0380	9
	0.1396	T20	0.1655	0.0231	13
	0.1396	T24	0.1067	0.0149	16
	0.1396	T19	0.0664	0.0093	17

Simultaneously, 12 experts were invited to evaluate the importance of the indicators (Table 17). Due to variations in the knowledge and experiences of each expert, assessments of the same two indicators could yield different results. The mode (most frequent value) was used to process the judgment matrix data to ensure data objectivity and fairness.

**Table 17:** Random consistency index.

n	1	2	3	4	5	6	7	8	9	10	11	12
RI	0	0	0.52	0.89	1.12	1.24	1.32	1.41	1.46	1.49	1.52	1.54

The weight values for all second-level demand indicators under the must-be quality indicators, one-dimensional quality indicators, and attractive quality indicators for Pingyao lacquerware tourism souvenirs were calculated by constructing comparison matrices. Their compliance with consistency testing standards was checked, where a smaller CR indicates better consistency of the judgment matrices. When CR < 0.1, the consistency of pairwise judgment matrices is within an acceptable range. However, when CR> 0.1, the judgment matrices demand to be reconstructed until the consistency ratio CR meets the demands. The test results are shown in Tables 12–15.

According to the formula, the weight result is: W1 = 0.527; W2 = 0.3325; W3 = 0.1396; Then the consistency test is carried out, and the maximum eigenvalue is obtained as follows *λ*_*max*_ = 3.0536, CI = (3.0536–3)/(3–1) = 0.0268, RI = 0.52, So, CR = $\frac{CI}{RI}$ = 0.0516<0.1, satisfying the consistency test.

According to the formula, the weight result is: (0.1693,0.1183,0.0720,0.2422, 0.3982); The maximum eigenvalue is *λ*_*max*_ = 5.1111, CI = 0.0278, CR = 0.0248<0.1, satisfying the consistency test.

According to the formula, the weight result is: (0.0797,0.0577,0.0568,0.3230, 0.2290,0.1430,0.1107); The maximum eigenvalue is *λ*_*max*_ = 7.2133, CI = 0.0356, CR = 0.0269<0.1, satisfying the consistency test.

According to the formula, the weight result is: (0.3893,0.0664,0.1655, 0.2721,0.1067); The maximum eigenvalue is *λ*_*max*_ = 5.1144,CI = 0.0286,CR = 0.0255<0.1, satisfying the consistency test.

In this study, the researchers classified and ranked the tourists’ demands through Kano model and AHP and got the following results.

1. Classified results:

1. Must-be quality: include Beautiful shape (T7), Nice pattern (T8), Nice color matching (T9), Quality can be guaranteed (T14), Environmentally friendly (T27).
2. One-dimensional quality: include Exquisite craft (T11), Reasonable price (T12), Rich types of products (T13), With multiple functions (T16), cultural taste (T17), Great commemorative significance (T22), Obvious regional characteristics (T23).
3. Attractive quality: include Lightweight and easy to carry (T15), Good packing (T19), Good brand (T20), Interesting idea (T21), Suitable as a gift (T24).
4. Indifferent quality consist of Many types of materials (T10), Have series (T18), Suitable for collection (T25), and High security (T26).

2. Ranked results (Table 18):

**Table 18:** 

Classification item	Sequence
	Must-be quality>One-dimensional quality>Attractive quality>Indifferent quality
Must-be quality	T27> T14 > T7 > T8 > T9
One-dimensional quality	T16 > T17 > T22 > T23 > T11 > T12 > T13
Attractive quality	T15 > T20 > T21 > T24 > T19
Appearance Quality	T7 > T8 > T9 > T11 > T10
Production Quality	T14 > T16> T17>T15>T12>T13 > T19> T18
Emotional Quality	T22 > T21 > T23 >T20 > T24 > T25
Extra Quality	T27 > T26

## Discussion and conclusion

This study utilized the KANO model to conduct an in-depth analysis of tourists’ demands for Pingyao lacquerware tourism souvenirs from four dimensions: appearance demands, production demands, emotional demands, and extra demands, providing targeted design ideas. Compared to existing research, this study offers a comprehensive analysis of tourists’ demands. It provides more precise guidance for designing lacquerware tourism souvenirs by classifying demand indicators and determining weight coefficients.

Firstly, tourists’ highest demand for lacquerware tourism souvenirs is for beautiful shape, followed by color and patterns. All these demands belong to essential demands, which are the basic requirements of tourists for lacquer souvenirs, and ignoring these demands will increase tourists’ dissatisfaction. Previous research has shown that people consistently pursue the shape, patterns, and colors of lacquerware products [41–43]. This study further verifies this and points out that under the backdrop of constantly changing tourist demands, the demand for product shapes has become more direct, providing new design focuses for designers. Therefore, designers should meet these demands as the minimum design standard and better grasp the importance of each design element.

In addition, this study found that besides appearance design demands, quality assurance and functional diversity of lacquerware are key demand factors, consistent with other related research [44, 45]. The variety of lacquerware products and reasonable pricing also show particular attractiveness. Tourists prefer lacquerware souvenirs with regional characteristics and commemorative solid significance. These demands are one-dimensional quality, reflecting competitiveness. Thus, these design points can effectively enhance tourists’ satisfaction with lacquerware tourism souvenirs, providing designers with different design ideas and thereby adding value to the souvenirs.

The study also found that although the importance of souvenirs’ portability has declined in the decision-making process, it still significantly enhances tourists’ satisfaction as an attractive quality. The absence of this demand will not reduce tourists’ satisfaction, but it can significantly increase it, offering a new perspective on souvenir design. Due to the uniqueness of lacquer materials, safety and environmental friendliness are also elements of particular concern to tourists, and they are becoming additional design focuses for lacquerware tourism souvenirs. This requirement also aligns with the concept of modern sustainable development design.

The presentation of this study is significant. It offers new design ideas for artisans and designers in the lacquerware souvenir industry and provides a reference for developing souvenir designs in other domestic tourist cities. It also deepens the understanding of Pingyao lacquerware, allowing more people to recognize its cultural value and significance, enhancing interest in lacquer culture, and aiding in preserving and inheriting traditional Pingyao lacquer art. Additionally, existing study has focused more on the craftsmanship and patterns of Pingyao lacquerware, with relatively fewer studies on the design of lacquerware souvenirs. This study attempts to fill this study gap.

Lastly, this study has certain limitations. Firstly, people’s demands vary, and tourists may have different demands for the same souvenir. This study can only explore the demands of most tourists in the tourist group and cannot cover every tourist’s demands. Secondly, demands change over time and may exhibit different characteristics under different economic backgrounds, therefore, the researcher’s findings regarding tourists demand only apply for a relatively short period. Furthermore, due to the uniqueness of lacquer materials and craftsmanship, there may be discrepancies between the design ideas and the actual production process of the souvenirs. Therefore, future designs for lacquerware souvenirs will require further study in conjunction with actual production situations.

## Supporting information

S1 Data (XLSX)
